# A31 IS AN ORAL SODIUM SULFATE SOLUTION MORE EFFECTIVE FOR PATIENTS WITH PRIOR CHALLENGING COLONOSCOPY PREPARATION? - A PROSPECTIVE CASE SERIES

**DOI:** 10.1093/jcag/gwae059.031

**Published:** 2025-02-10

**Authors:** A Khan, M Rai, J McKay, R Mohanna, L Hookey

**Affiliations:** Internal Medicine, Kingston Health Sciences Centre, Kingston, ON, Canada; Gastrointestinal Diseases Research Unit, Queen’s University, Kingston, ON, Canada; Gastrointestinal Diseases Research Unit, Queen’s University, Kingston, ON, Canada; Gastrointestinal Diseases Research Unit, Queen’s University, Kingston, ON, Canada; Gastrointestinal Diseases Research Unit, Queen’s University, Kingston, ON, Canada

## Abstract

**Background:**

A considerable proportion of patients encounter difficulties with existing bowel cleansing regimens for colonoscopy resulting in suboptimal bowel visualization. This impacts patients, endoscopists, those on the waiting list, and the endoscopy unit overall.

**Aims:**

Oral Sulfate Solution (OSS) has been proven to be efficacious in many situations and therefore this study aims to explore its benefit in patients who had challenging preparations during their previous colonoscopy.

**Methods:**

This prospective case series was conducted at Kingston Health Sciences Centre. Patients scheduled for outpatient colonoscopy were considered for enrolment if previous colonoscopy reports indicated criteria of suboptimal preparation. Participants were allowed a light breakfast the day before the colonoscopy and consumed the first dose of OSS in a split dose fashion. The primary outcome was adequacy of bowel preparation using the Boston Bowel Preparation Scale (BBPS), defined as an overall score greater than 6, with each segment scoring at least 2. Secondary outcome measures included time to cecum, volume suctioned, and tolerability rating.

**Results:**

A total of 120 patients with colonoscopies that were deemed as challenging to prepare were included in this study. Inclusion criteria for 109 of these patients was documentation of extensive washing and suctioning for adequate view of the colon, 5 had poor or fair preparation with images revealing obvious debris and 6 either vomited or consumed 80% or less of the preparation. Participants mean age was 67 years (SD=7.151), 66 (55%) were male and 24 (20%) had diabetes. The average BBPS score was 7 (SD=1.757) and 88% of the patients had adequate preparation. Participants’ prior colonoscopy preparations were primarily polyethylene glycol (52.5%) and a picosulfate based agent with bisacodyl (40.8%). Mean time to cecum was 7 minutes and 29 seconds (SD= 4 minutes and 29 seconds). The mean total volume suctioned was 1048.78mls (SD= 708.621). Tolerability data demonstrated 107 (89.2%) participants cumulatively rated OSS overall as tolerable, easy or very easy to use. Nausea was reported by 46 participants (38.3%), while vomiting occurred in 10 (8.3%). Abdominal distension or bloating was noted by 34 participants (28.3%), and 51(42.5%) experienced abdominal cramping, discomfort, or pain.

**Conclusions:**

OSS showed a reduction to 12% inadequate bowel preparation. This is very encouraging given this group of patients had challenging preparation in the past. Its combination of effect, ease of use, and tolerability could deem it superior to traditional choice in these patients and can be used as a standard low volume preparation.

**Funding Agencies:**

Pendopharm

